# circRAE1 promotes colorectal cancer cell migration and invasion by modulating miR-338-3p/TYRO3 axis

**DOI:** 10.1186/s12935-020-01519-x

**Published:** 2020-09-03

**Authors:** Jiabin Du, Jianhua Xu, Junxing Chen, Weinan Liu, Pengcheng Wang, Kai Ye

**Affiliations:** Department of Gastrointestinal Surgery, Second Affiliated Hospital of Fujian Medical University, No.950 Donghai Dajie, Fengze District, Quanzhou, 362000 Fujian China

**Keywords:** circRNAs, Colorectal cancer, circRAE1, miR-338-3p, TYRO3

## Abstract

**Background:**

Growing evidence has revealed the involvement of circular RNAs (circRNAs) in numerous carcinogenesis. However, the role of circRNAs in the cancer biology of colorectal cancer (CRC) remains vague.

**Methods:**

Quantitative RT-PCR was used to detect the expression level of circRAE1 in CRC tissues and CRC cell lines. Cell proliferation, migration, and invasion were detected using CCK8 assay, Colony formation assay, wound-healing and Transwell assays. The interaction between circRAE1 and miR-338-3p and TRYO3 was confirmed using dual-luciferase reporter assays.

**Results:**

We uncovered a novel circRNA Hsa_circ_0060967 (also known as circRAE1) that was remarkably increased in CRC tissues. The high circRAE1 level was positively associated with advanced tumor stage, lymph node metastasis, and tumor size. The loss-of-function assay showed that circRAE1 accelerated cell proliferation, migration, and invasion. Besides, miR-338-3p was lowly expressed in the CRC tissues and CRC cell lines. The dual-luciferase reporter assays showed that circRAE1 could sponge miR-338-3p, which targeted TRYO3 in CRC cells. Furthermore, the overexpression of circRAE1 could rescue the impaired migration and invasion triggered by miR-338-3p mimics or si-TYRO3 in CRC cells and vice versa.

**Conclusion:**

We identified the network of circRAE1, miR-338-3p, and TYRO3 in CRC cells and determined that the increase in circRAE1 could serve as an oncogene by sponging miR-338-3p, which resulted in an upregulated TYRO3 expression. The finding suggests that circRAE1 is a potential therapeutic target and diagnostic marker for CRC treatment.

## Background

Colorectal cancer (CRC) is one of the most commonly diagnosed cancers worldwide, leading to more than 600,000 deaths each year [1, 2]. Approximately 25% of patients with CRC were reported to have distant metastasis at the time of diagnosis. In addition, 25% of the patients experienced metastasis during treatment, and the prognosis of advanced patients was poor, with a 5-year survival rate for less than 15% of them [1]. With the advances in the diagnosis and treatment of CRCs, the 5-year relative survival rates of patients with stage I or II disease have increased to 91% and 82%, respectively [3]. However, the 5-year survival rate decreased to 12% among patients suffering distant metastasis [3]. Therefore, further clinical research is needed to improve the 5-year survival rate of CRC patients with distant metastasis as a means of enhancing treatment efficiency, and the molecular mechanisms of CRC tumorigenesis and its novel targets should be understood and identified, respectively.

The circular RNA (circRNA) is a non-coding RNA characterized by its stable circularized shape, and it is pervasively expressed in various cell types [4, 5]. These circRNAs are resistant to exonuclease-mediated degradation and usually serve as competing endogenous RNAs (ceRNAs) to reduce downstream microRNA levels and function [6, 7]. Recently, growing evidence has shown that circRNAs are involved in the regulation of tumorigenesis. Su et al. found that hsa_circ_0070269 suppresses hepatocellular carcinoma (HCC) cell proliferation and invasion and inhibits HCC tumor growth in vivo by sponging miR-182 to promote NPTX1 translation in HCC cells [8]. Li et al. showed that circ-ITCH, when decreased in esophageal squamous cell carcinoma, plays an antitumor role and acts as a ceRNA by sponging miR-17, miR-214, and miR-7 to increase ITCH mRNA [9]. Kong et al. revealed the hsa_circ_0085131/miR-654-5p/ATG7 axis as a potential therapeutic option among non-small cell lung cancer (NSCLC) patients who are resistant to cisplatin [10]. Moreover, emerging studies have shown that circRNAs are involved in the tumorigenesis and metastasis of CRC and identified as latent therapeutic targets for CRC treatment [11]. Chen et al. reported that the circ-001971/miR-29c-3p axis modulates CRC cell proliferation, invasion, and angiogenesis by targeting VEGFA [12]. Ma et al. showed that circ5615 exerts an oncogenic function as the ceRNA of miR-149-5p to release TNKS and activated Wnt/β-catenin pathways [13]. However, the detailed roles of circRNAs in CRC tumorigenesis and metastasis are only beginning to be revealed.

We identified a novel circRNA (also known as hsa_circ_0060967 but renamed as circRAE1 in this study) that was remarkably increased in CRC tissues. A high circRAE1 level was positively associated with advanced tumor stage, tumor size, and lymph node metastasis. Additionally, we confirmed that circRAE1 played an oncogenic role in the progression of CRC by serving as a ceRNA for miR-338-3p to increase TYRO3 expression. The findings suggest that circRAE1 is a novel potential therapeutic target for CRC treatment intervene.

## Materials and methods

### CRC patients and clinical tumor tissues

Eighty pairs of CRC tissues and the adjacent non-tumor tissues were collected from the Second Affiliated Hospital of Fujian Medical University in January–December 2019. The collection was approved by the Ethical Committee of the Second Affiliated Hospital of Fujian Medical University, and all patients were informed of the research details before admission. No additional treatment was given to the patients before surgery. The tissues were snap-frozen in liquid nitrogen and stored at − 80 °C.

### Cell culture and transfection

The human CRC cells HCT116, SW620, HT29, and SW480 and the normal colonic epithelial cell lines NM460 and HEK293T were obtained from the Cell Bank of Chinese Academy of Sciences (Shanghai, China). All cells were cultured in DMEM-high glucose (Gibco) containing 10% fetal bovine serum (PAN Biotech) and 100 u/mL penicillin/streptomycin at 37 °C under 5% CO_2_ in a humidified chamber.

The miR-338-3p mimics, miR-negative control (miR-NC), siRNAs for circRAE1 (si-circRAE1#1, 2, and 3), TYRO3 (si-TYRO3), and siRNA-negative control (si-NC) were obtained from Shanghai Gene-Pharma. The circRAE1-overexpression plasmid was constructed using the Pcd-ciR vector. All recombinant plasmids were sequenced and confirmed by Fuzhou Biosune. Then, the circRAE1-overexpression plasmid was co-transfected with RRE, REV, and VSV/G into HEK293T cells to produce lentivirus-circRAE1 (lv-circRAE1). The si-circRAE1 (100 nM), si-TYRO3 (100 nM), and miR-338 mimics (50 nM) were transfected using the Lipofectamine 2000 reagent (Life) according to product specification. The lentivirus-circRAE1 was infected with CRC cells and 10 μg/mL polybrene. The cell, RNA, and protein samples were collected 24 or 48 h after transfection.

### Quantitative RT-PCR (qRT-PCR) assays

Total RNA was isolated with the TRIzol reagent according to product specification. The cDNA was synthesized using a reverse transcription kit (Sangon, China) for circRNA and mRNA analysis. The RiboBio microRNA reverse transcription kit (Guangzhou, China) was used for the miRNA. Quantitative PCR was conducted using GoTaq^®^ qPCR Master Mix (Promega, USA). The relative expression levels of circRNAs, mRNAs, and miRNAs were calculated as the method of 2^−ΔΔCt^ and normalized to GAPDH for circRNAs and mRNAs or U6 for miRNAs. Each experiment was carried out in triplicate. The primer sequences are shown in Table 1.

**Table 1:** Sequences used for qRT-PCR and siRNA assays

Names	Sequences (5′— 3′)	
circRAE1	Forward	AGAGAGGGGCCTGATTGTCTA
	Reverse	AGTGTGCATCTGCTGATGTTTC
E-cadherin	Forward	GCTGGACCGAGAGAGTTTCC
	Reverse	CAAAATCCAAGCCCGTGGTG
Vimentin	Forward	GGACCAGCTAACCAACGACA
	Reverse	AAGGTCAAGACGTGCCAGAG
Linear RAE1	Forward	AGGCAGTACTTCTCCAGGTTCA
	Reverse	GGCAAGGTTGGTGGGCTAAA
TYRO3	FORWARD	AGTTGGCTGTGGACCCTGGAG
	REVERSE	AGGATGTGCGGCTGTGAGGAG
GAPDH	Forward	ATGGGGAAGGTGAAGGTCG
	Reverse	TTACTCCTTGGAGGCCATGTG
miR-338-3p	Forward	CGCGTCCAGCATCAGTGATT
	Reverse	AGTGCAGGGTCCGAGGTATT
	RT Primer	GTCGTATCCAGTGCAGGGTCCGAGGTATTCGCACTGGATACGACCAACAA
U6	Forward	CTCGCTTCGGCAGCACATATACT
	Reverse	ACGCTTCACGAATTTGCGTGTC
	RT Primer	AAAATATGGAACGCTTCACGAATTTG
si-circRAE1-Homo1	Sense	GGAUCAAAGCUCCAAACUACA
	Antisense	UAGUUUGGAGCUUUGAUCCAA
si-circRAE1-Homo2	Sense	GCAGUGCAACUACAGACAAUC
	Antisense	UUGUCUGUAGUUGCACUGCCA
si-circRAE1-Homo3	Sense	GGACCUCAGCAGUAACCAAGC
	Antisense	UUGGUUACUGCUGAGGUCCCA
si-TYRO3	Sense	GGUGGAGAGGAACUACGAAGA
	Antisense	UUCGUAGUUCCUCUCCACCAG
Negtive control	Sense	AUGGAAUUUUGUUGUGAAAUC
	Antisense	UUCACAACUUAAUUCCAAGAU

### Western blot assay

Western blot assays were conducted as previously described [13]. Briefly, equal amounts of proteins were separated on 10%–12% SDS-PAGE, transferred onto PVDF membranes (0.45 μM), and blocked with 5% skim milk. Then, the membranes were incubated with various primary antibodies at 4 °C for more than 10 h. Afterwards, the membranes were blotted with HRP-conjugated secondary antibodies. The signal was visualized with ECL (Beyotime Biotechnology, Jiangsu, China) using the LiCor C-DiGit Blot scanner with the LiCor Biosciences Image Studio software. The anti-E cadherin antibody (HECD-1) (1:1000), anti-vimentin antibody (RV202) (1:1000), and anti-TYRO3 antibody (EPR4308) (1:1000) were obtained from Abcam.

### Stability analysis of circRAE1

The SW620 and HT29 were treated by actinomycin D (2 μg/mL), then total RNA was extracted after 0, 4, 8, 12, and 24 h. The levels of circRAE1 and its linear subtype were detected by qRT-PCR. As for the RNase digestion assay, its total RNA was co-incubated with three units of RNase R for every 1 μg of RNA for 30 min at 37 °C. Then, the re-purified RNA was subjected to qRT-PCR to determine the circRAE1 and its linear subtype.

### Fluorescence in situ hybridization

In situ hybridization was employed to explore the intracellular location of circRAE1 and miR-338-3p in HT29 and SW620. The cells were grown on cover slips and fixed with 4% paraformaldehyde (PFA). The procedure was carried out as previously reported [14]. Briefly, the slides were treated with CSK buffer (0.5% TritonX-100, 10 mM VRC) for 10–12 min, then treated with 70% alcohol for 10 min at 4 °C, followed by incubation with series alcohol for dehydration. After air drying, the slides were prehybridized for 1 h at 55 °C in a prehybridization solution. The Cy3-circRAE1 and/or digoxin-labeled miR-338-3p (DIG-miR338-3p) probe in the hybridization buffer were denatured at 76 °C for 10 min, then added to each slide, and hybridized overnight at 37 °C in a dark humidified chamber. After washing, the anti-DIG [21H8]-FITC (Abcam, USA) was added to the slides, and the slides were incubated at 37 °C for 1 h in the humidified chamber. Finally, the slides were counterstained with 4,6-diamidino-2-phenylindole (DAPI) after washing.

### Luciferase reporter assay

The PCR method was used to amplificate the wild-type (WT) and mutant (MUT) 3′-UTR of the human TYRO3. Then, the WT and MUT of circRAE1 were cloned into the psiCHECKTM-2-luciferase reporter plasmid. The HEK293T cells were co-transfected with WT or MUT psiCHECKTM-2-circRAE1 plasmids or psiCHECKTM-2-TYRO3 (3′-UTR) and miR-338-3p mimics or miR-NC by using the Lipofectamine 2000 reagent. After 48 h, the cells were collected and subjected to the commercial Dual-Luciferase reporter assay system (Promega) following the manufacturer’s instructions for measuring firefly and Renilla luciferase activities.

### CCK-8 assay

The cell proliferation rates were detected with the Cell Counting Kit-8 (CCK-8, Dojindo, Japan). The 1 × 10^4^ cells were cultured in each 96-well plate for 0, 24, 48, and 72 h. Subsequently, the absorbance at 450 nm was measured after incubation with 10 μL 10% CCK-8 solution at 37 °C for 1–2 h. All detections were carried out in triplicate.

### Colony formation assay

SW620 and HT29 cells (800 for each well) were seeded in six-well plates for two weeks. Subsequently, the colonies were stained with 1% crystal violet for 10 min after fixation with 4% paraformaldehyde for 5 min. The colonies were microscopically examined and counted. The assays were repeated three times.

### Wound-healing assay

A wound migration model for the in vitro assay was used as previously described [15]. Culture inserts (Ibidi) were used in the wound-healing assay. Briefly, HT29 and SW620 cells were seeded to each well of the culture inserts. The cells were incubated at 37 °C for 24 h, and a cell-free gap of 500 μm was created after the removal of the culture insert. An inverted phase-contrast microscope was used to capture the images at 0, 24, and 48 h. Five randomly chosen fields were used to calculate the percentage of wound closure using the ImageJ software.

### Transwell migration assay

Transwell chambers (Corning, USA) were used in the Transwell migration assay as previously described [9]. The SW620 and HT29 cells were seeded into the upper chambers with serum-free DMEM, while the lower wells were filled with DMEM containing 20% FBS. After 24 h, the cells in the top chamber were removed with cotton swabs, while those cells in the lower surface were fixed using 4% PFA and stained with 0.1% crystal violet for 15 min. After washing twice with PBS, the cell numbers in five randomly chosen fields were calculated under a microscope (Olympus, Japan).

### Transwell invasion assay

The Transwell invasion assay was detected as previously described [16]. Briefly, the upper chambers (Corning, USA) were coated with diluted Martrigel (BD, USA). Afterwards, the SW620 and HT29 cells were seeded into the upper chambers with serum-free DMEM, then the Transwell chambers were incubated in wells filled with DMEM containing 10% FBS. After 24 h of incubation at 37 °C, the cells in the inner chambers and the remaining Matrigel were removed with cotton swabs, while the cells on the outside surface of the lower chambers were fixed using 4% PFA and stained with 0.1% crystal violet for 15 min. After washing twice with PBS, the cell numbers in the five randomly chosen fields were calculated under a microscope.

### Statistical analysis

The SPSS 22.0 statistical software was used for the statistical analysis. The data were calculated as mean ± the standard deviation (SD). The significant differences between groups were estimated using one-way ANOVA. *P* < 0.05 was considered statistically significant.

## Results

### circRAE1 was upregulated in CRC tissues and CRC cell lines

**Fig. 1:**
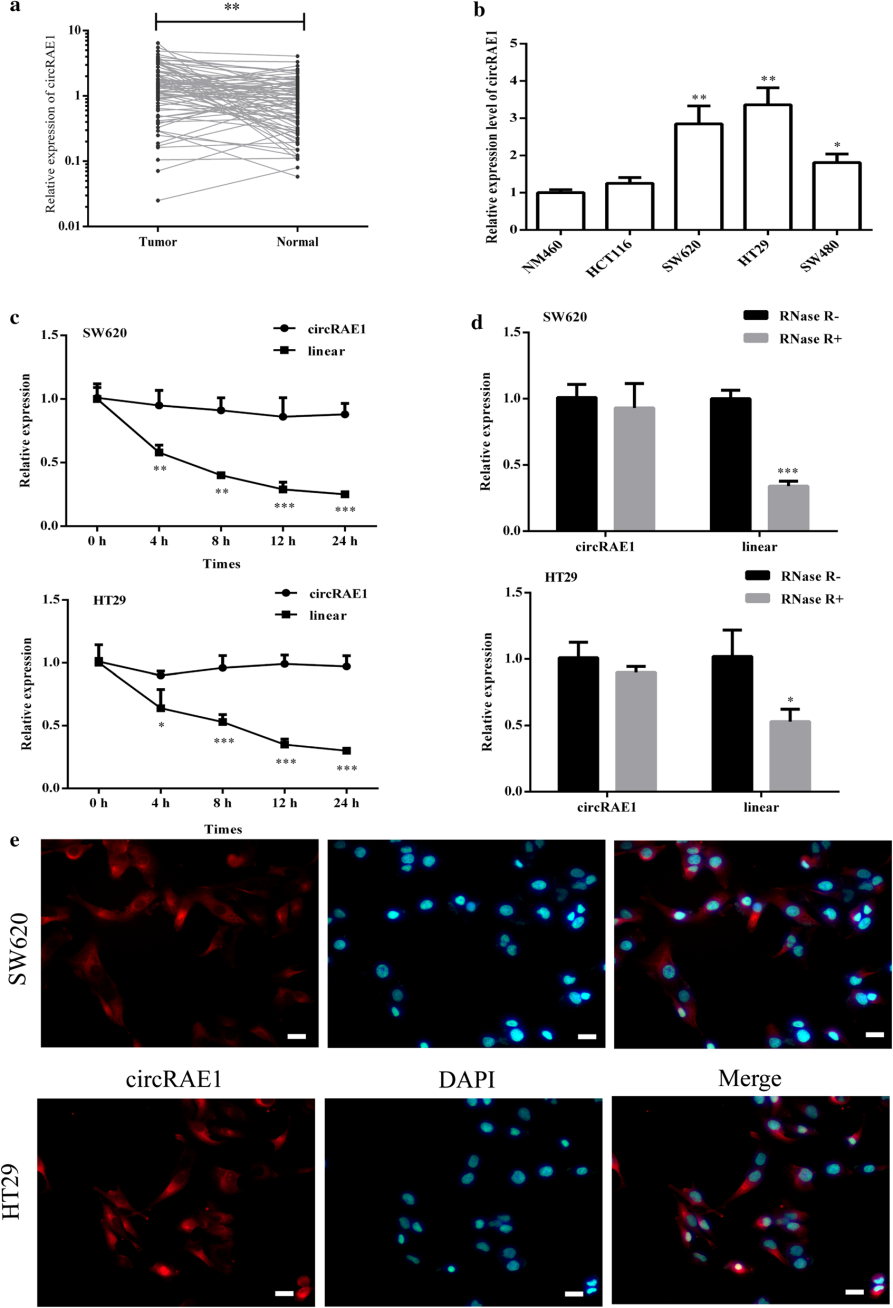
CircRAE1 was upregulated in CRC. **a** Expression of CircRAE1 in paired CRC tissues from 80 CRC patients measured by qRT-PCR. **b** Relative expression levels of CircRAE1 in normal cell line (NM460) and CRC cell lines (HCT116, SW620, HT29, and SW480) by qRT-PCR. **c** Relative expression level of CircRAE1 and its linear types in SW620 and HT29 cells at various time points after treatment by actinomycin D. **d** Total RNA from SW620 or HT29 cells treated with RNase R. CircRAE1 was stably expressed in both RNase R(−) and RNase R(+) total RNA samples. **e** RNA in situ hybridization for CircRAE1 (red) in sw620 and HT29 cells (× 200). Nuclei were stained with DAPI (blue). Scare bar = 100 μm.*P < 0.05, **P < 0.01, ***P < 0.001

A previous study based on CapitalBio microarray data showed that numerous circRNAs were differentially expressed in CRC tissues unlike their normal adjacent tissues [17]; among them, the hsa_circ_0060967 (the corresponding gene symbol is RAE1 and thus renamed as circRAE1 in this study) was significantly upregulated. As the expression properties of circRAE1 in the CRC required validation, the circRAE1 expression in the 80 paired CRC tissues were detected using qRT-PCR. The circRAE1 expression was upregulated in the CRC tissues (Fig. 1a). On the basis of the analyzed clinical data and the expression levels of circRAE1, we can conclude that high circRAE1 expressions are positively related to advanced TNM stage, large tumor size, and lymph node metastasis (Table 2).

**Table 2:** The relationship analysis between relative expression levels of circRAE1 in CRC cancerous tissues and clinical characteristics

Clinical characteristics	Total (n = 80)		CircRAE1 expression		
	Counts		Low	High	P value
Gender	Male	46	17	29	0.1143
	Female	34	7	27	
Age	≤ 65	49	13	36	0.3946
	> 65	31	11	20	
Tumor size	< 5 cm	50	19	31	0.0438
	≥ 5 cm	30	5	25	
Tumor site	Colon	25	9	16	0.4298
	Rectum	55	15	40	
Depth of invasion	T1–T2	10	5	5	0.1401
	T3–T4	70	19	51	
Lymph node metastasis	N0	37	20	17	< 0.001
	N1–N2	43	4	39	
Distant metastasis	M0	78	24	54	0.3484
	M1	2	0	2	
TNM stage	I–II	37	20	17	< 0.001
	III–IV	43	4	39	

We then explored the circRAE1 expression in CRC cells and found that the circRAE1 expression levels were significantly higher in CRC cell lines (SW620, HT29, and SW480) compared with the normal colonic epithelial cell line NM460 (Fig. 1b), especially for SW620 and HT29. Subsequently, we explored the stability of circRAE1 by adding actinomycin D, which can block new transcription, and detected the levels of circRAE1 and its linear control by qRT-PCR. The analyzed data showed that circRAE1 was more stable than the linear mRNA (Fig. 1c). Moreover, we observed that circRAE1 was resistant to RNase R, whereas its linear subtype was not resistant (Fig. 1d). In addition, the fluorescence in the in situ hybridization results showed that circRAE1 was dominantly localized in the cytoplasm (Fig. 1e).

### circRAE1 increased CRC cell migration and invasion ability

**Fig. 2:**
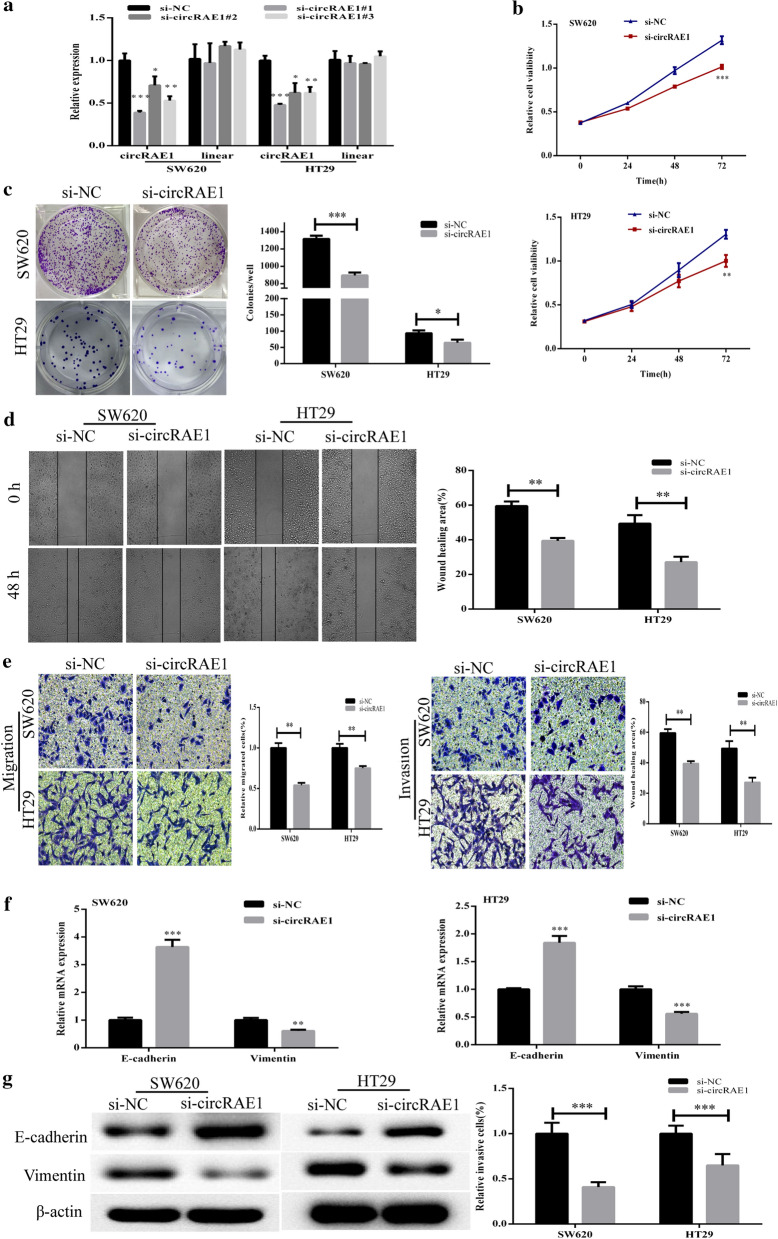
Function of CircRAE1 in CRC cells. **a** Expression of CircRAE1 after transfection with various siRNAs and negative control for 48 h in SW620 and HT29 cells as detected by qRT-PCR. **b**, **c** Downregulation of CircRAE1-suppressed SW620 and HT29 cell proliferation ability as determined by CCK8 and colony formation assays. **d** Downregulation of CircRAE1-suppressed SW620 and HT29 cell migration ability as determined by wound healing tests. **e** Downregulation of CircRAE1-suppressed SW620 and HT29 cell migration and invasion ability as determined by Transwell assays. **f** Expression of E-cadherin and vimentin in SW620 and HT29 as detected by qRT-PCR after transfection with si-circRAE1 or si-NC. **g** Protein level of E-cadherin and vimentin in SW620 and HT29 as detected by Western blot assay. *P < 0.05, **P < 0.01, ***P < 0.001

Here, we intended to select SW620 and HT29 for certain functional assays in consideration of the high-expression levels of circRAE1 in the two cell lines (Fig. 1b). We transfected the cells with various siRNAs by targeting circRAE1 and found that all the three siRNAs could effectively knock down circRAE1 expression, in which si-circRAE1#1 attained the best interference efficiency (Fig. 2a). Thus, we selected si-circRAE1#1, which we named si-CircRAE1, for the succeeding circRAE1 knockdown assays. The CCK8 and colony assays presented a silencing of circRAE1 that could markedly inhibit proliferation in the two cell lines (Fig. 2b, c). The scratch tests showed a downregulation of circRAE1, which significantly reduced the wound healing efficiencies of the SW620 and HT29 cells (Fig. 2d). Subsequently, the Transwell assays showed that the downregulation of circRAE1 significantly inhibited SW620 and HT29 cell migration and invasion activities in vitro (Fig. 2e). Furthermore, we explored whether circRAE1 would be involved in the epithelial–mesenchymal transition (EMT) process. Thus, the expression of the EMT marker genes (E-cadherin and vimentin) were detected, and the circRAE1 expression was silenced (Fig. 2f). Both the mRNA and protein levels of E-cadherin was remarkably upregulated by the knocking down of circRAE1, whereas vimentin presented the reverse (Fig. 2g). These results indicate that circRAE1 may serve as an oncogene in CRC progression by regulating the expression of EMT markers.

### circRAE1 targeted miR-338-3p

**Fig. 3:**
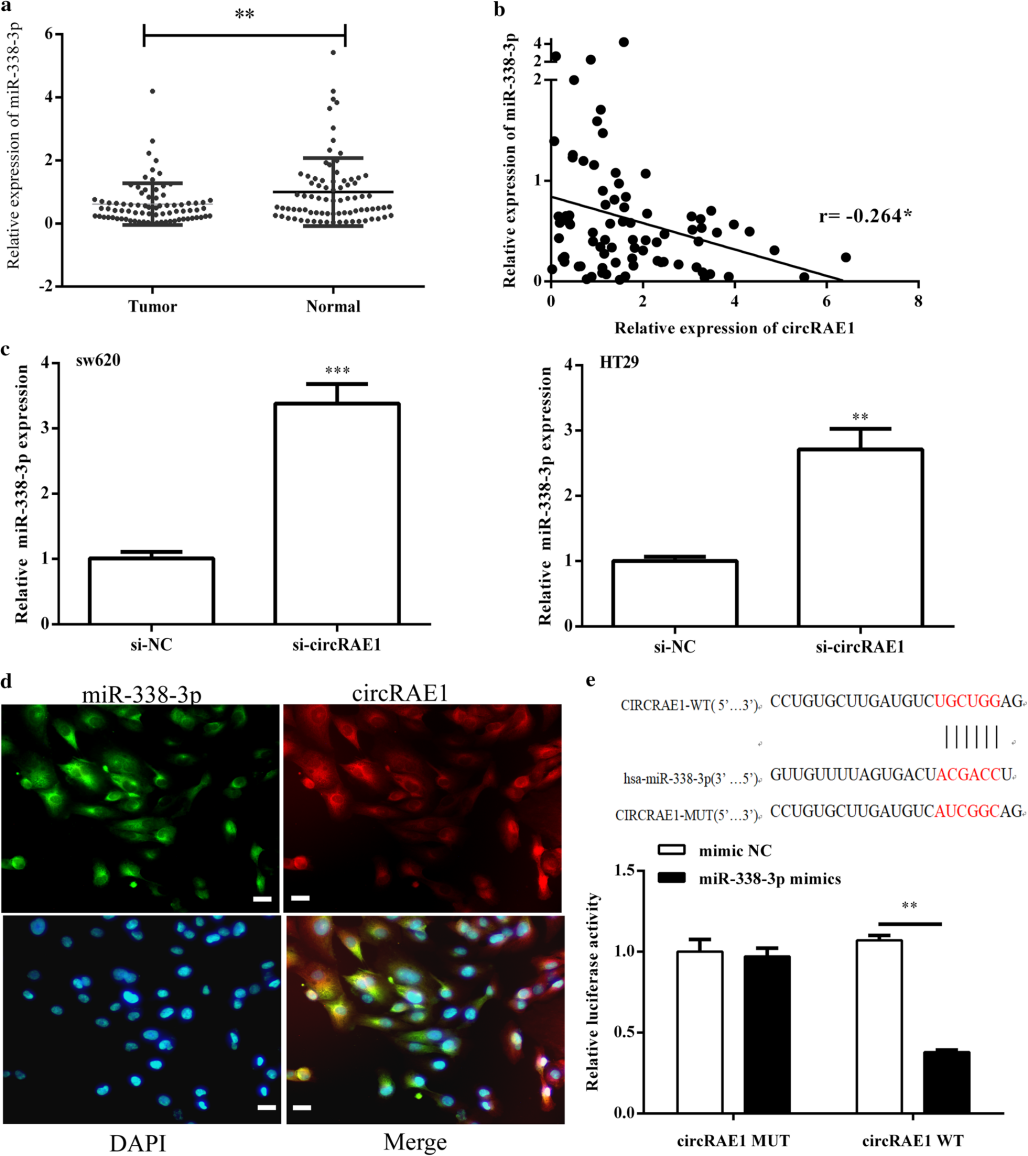
CircRAE1 serves as ceRNA for miR-338-3p in CRC cells. **a** Expression of miR-338-3P in paired CRC tissues from 80 CRC patients as measured by qRT-PCR. **b** CircRAE1 expression that is negatively correlated with miR-338-3P expression in CRC tissues. **c** Downregulation of CircRAE1-promoted miR-338-3p expression in SW620 and HT29 cells as determined by qRT-PCR. **d** RNA in situ hybridization for miR-338-3p (green) and CircRAE1 (red) in SW620 cells (× 200). Nuclei were stained with DAPI (blue). Scare bar = 100 μm **e** Significantly reduced luciferase activity of CircRAE1 WT by miR-338-3p mimics. *P < 0.05, **P < 0.01, ***P < 0.001

The ceRNA, as a newly proposed mechanism, entails the crosstalk among lncRNAs, including circRNA, mRNAs, and their shared miRNAs. By using an online tool (CircInteractome), we speculate that the potential miRNAs may be bound by CircRAE1. Among them was miR-338-3p, which was dramatically reduced in CRC tissues (Fig. 3a), and it showed a negative relation with CircRAE1 in the expression (Fig. 3b). In addition, by using si-CircRAE1, we determined that downregulation of CircRAE1 could notably increase the miR-338-3p expression in SW620 and HT29 cells (Fig. 3c). Furthermore, the fluorescence in situ hybridization results indicate that CircRAE1 and miR-338-3p were co-localized in the cytoplasm (Fig. 3d). The direct binding of miR-338-3p to circRAE1 was further confirmed. We constructed circRAE1 WT and circRAE1 MUT luciferase reporter plasmids. The luciferase reporter assay showed that the co-transfection with miR-338-3p mimics repressed the luciferase activity of circRAE1 WT, whereas the circRAE1 MUT was not affected by the co-transfection (Fig. 3e). This finding indicates that circRAE1 can directly sponge miR-338-3p.

### circRAE1 functions by targeting miR-338-3p

**Fig. 4:**
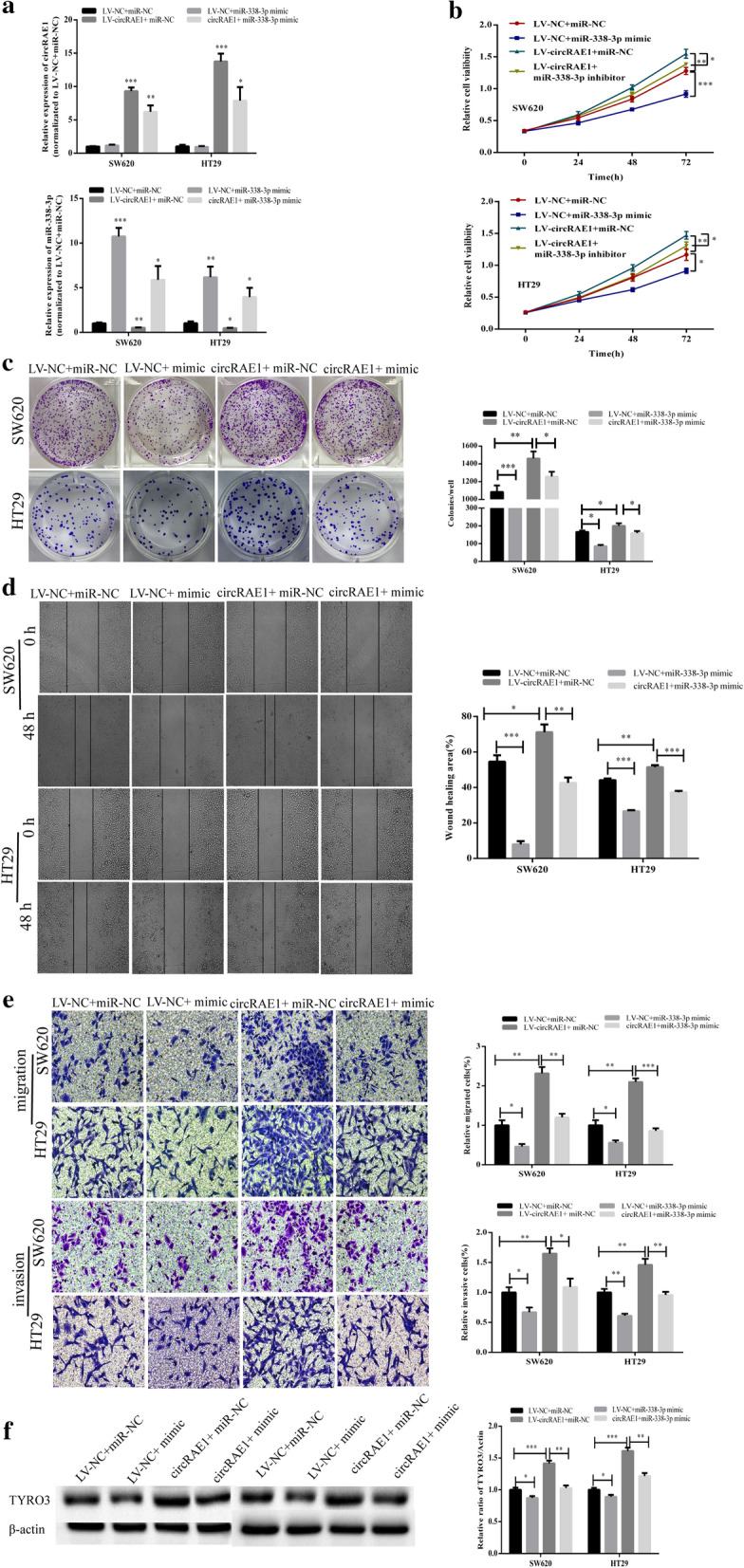
CircRAE1 serves as an oncogene by sponging miR-338-3p in CRC cells. **a** Non-significant alteration by miR-338-3P mimics of the relative expression of miR-338-3p or Significant reduction by the overexpression of CircRAE1 with LV-CircRAE1 of the miR-338-3p expression in SW620 and HT29 cells as determined by qRT-PCR. **b**, **c** Dramatic rescue by the overexpression of CircRAE1 of the cell proliferation ability of SW620 and HT29 cells induced by miR-338-3p mimics and vice versa. **d** Dramatic rescue by the overexpression of CircRAE1 of the impaired wound healing ability of SW620 and HT29 cells induced by miR-338-3p mimics and vice versa. **e** Dramatic rescue by the overexpression CircRAE1 of the impaired migration and invasion ability of SW620 and HT29 cells induced by miR-338-3p mimics and vice versa as detected by Transwell assays. **f** Protein level of TYRO3 in SW620 and HT29 as detected by Western blot assay. *P < 0.05, **P < 0.01, ***P < 0.001

First, in exploring the function of circRAE1 bound by miR-338-3p, we determined whether the circRAE1 overexpression could alter the miR-338-3p expression or whether the miR-338-3p mimic could affect the circRAE1 expression. The results suggest that the miR-338-3p mimic had no effect on the circRAE1 expression, while the circRAE1 overexpression remarkably reduced miR-338-3p (Fig. 4a), further indicating that circRAE1 could control miR-338-3p expression. Additionally, we employed wound CCK8, colony assays, healing assay, and Transwell migration and invasion experiments to determine if miR-338-3p influenced CRC cell migration and invasion through the miR-338-3p. As shown in Fig. 4b–e, the miR-338-3p mimic suppresses and inhibits the proliferation, and the wound closure in the SW620 and HT29 cells and the circRAE1 overexpression have partially reversed the effects caused by the miR-338-3p mimics on both cells. The migration and invasion capacity of the SW620 and HT29 cells, as determined by the Transwell assays, was also inhibited by the miR-338-3p mimic and rescued by the circRAE1 overexpression (Fig. 4e).We found that the TYRO3 protein level was upregulated by LV-CircRAE1 (Fig. 4f).

### TYRO3 was directly targeted by miR-338-3p

**Fig. 5:**
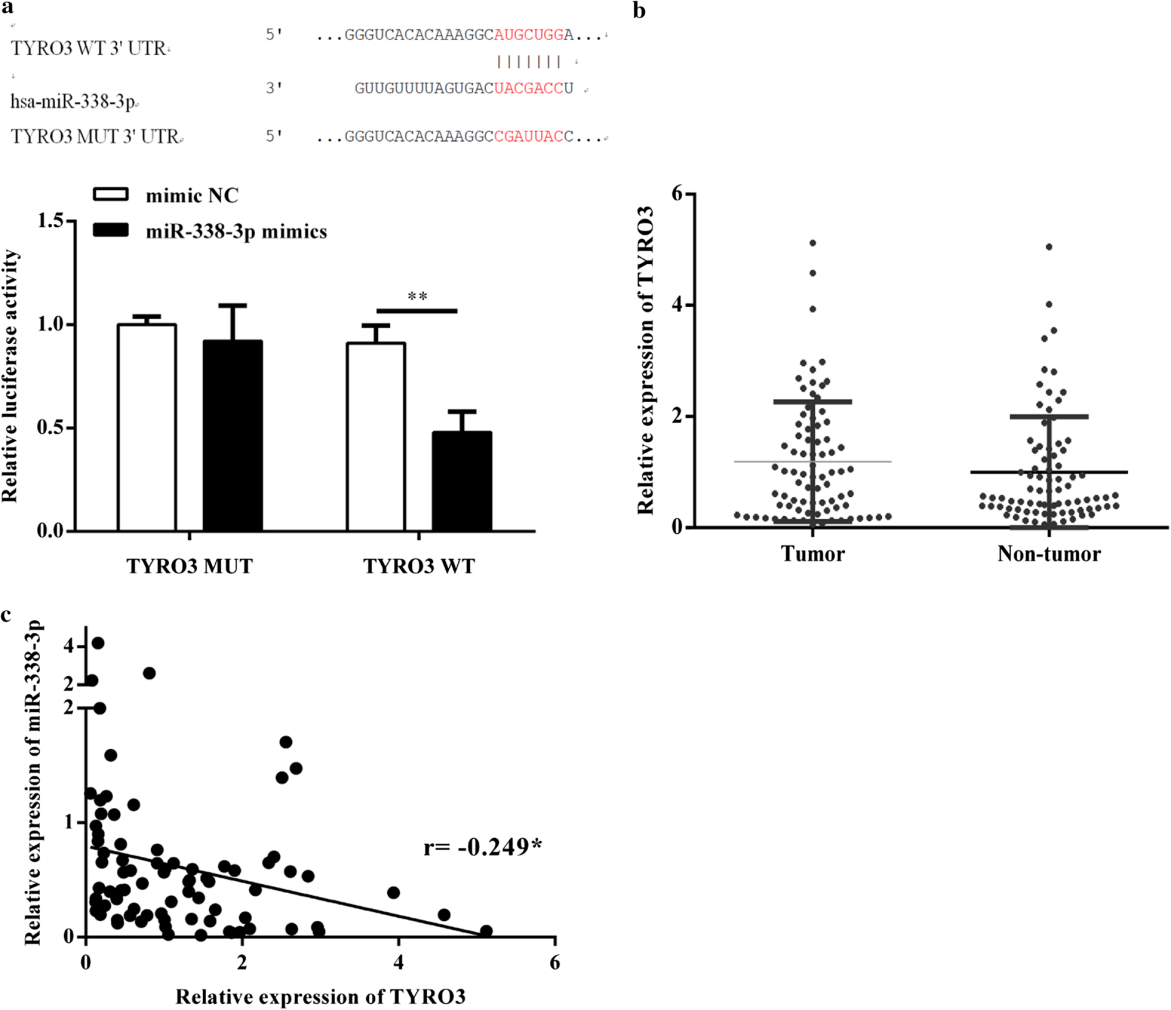
TYRO3 was a direct target gene of miR-338-3p. TYRO3 axis in CRC. **a** Binding site between miR-338-3p and the 3′-UTR of TYRO3. **b** Significant reduction by the miR-338-3p mimics of the luciferase activity of TYRO3 WT. **c** Expression of TYRO3 in paired CRC tissues from 80 CRC patients as measured by qRT-PCR. **P < 0.01

The bioinformatics analysis, which was carried out using Targetscans (https://www.targetscan.org/), showed that TYRO3 was a putative target of miR-338-3p. The binding sites in between is shown in Fig. 5a. Then, the luciferase reporter plasmids of TYRO3-WT and TYRO3-MUT were constructed in our study (Fig. 5a). The luciferase reporter assay showed that adding miR-338-3p mimic could dramatically inhibit the reporter activity of TYRO3-WT rather than TYRO3-MUT (Fig. 5a), confirming that miR-338-3p could directly bind to the 3′-UTR of TYRO3. Moreover, the expression level of TYRO3 was also notably upregulated in CRC tissues (Fig. 5b) and showed a negative relation with CircRAE1 in the expression (Fig. 5c).

### circRAE1 functions by regulating TYRO3 expression

**Fig. 6:**
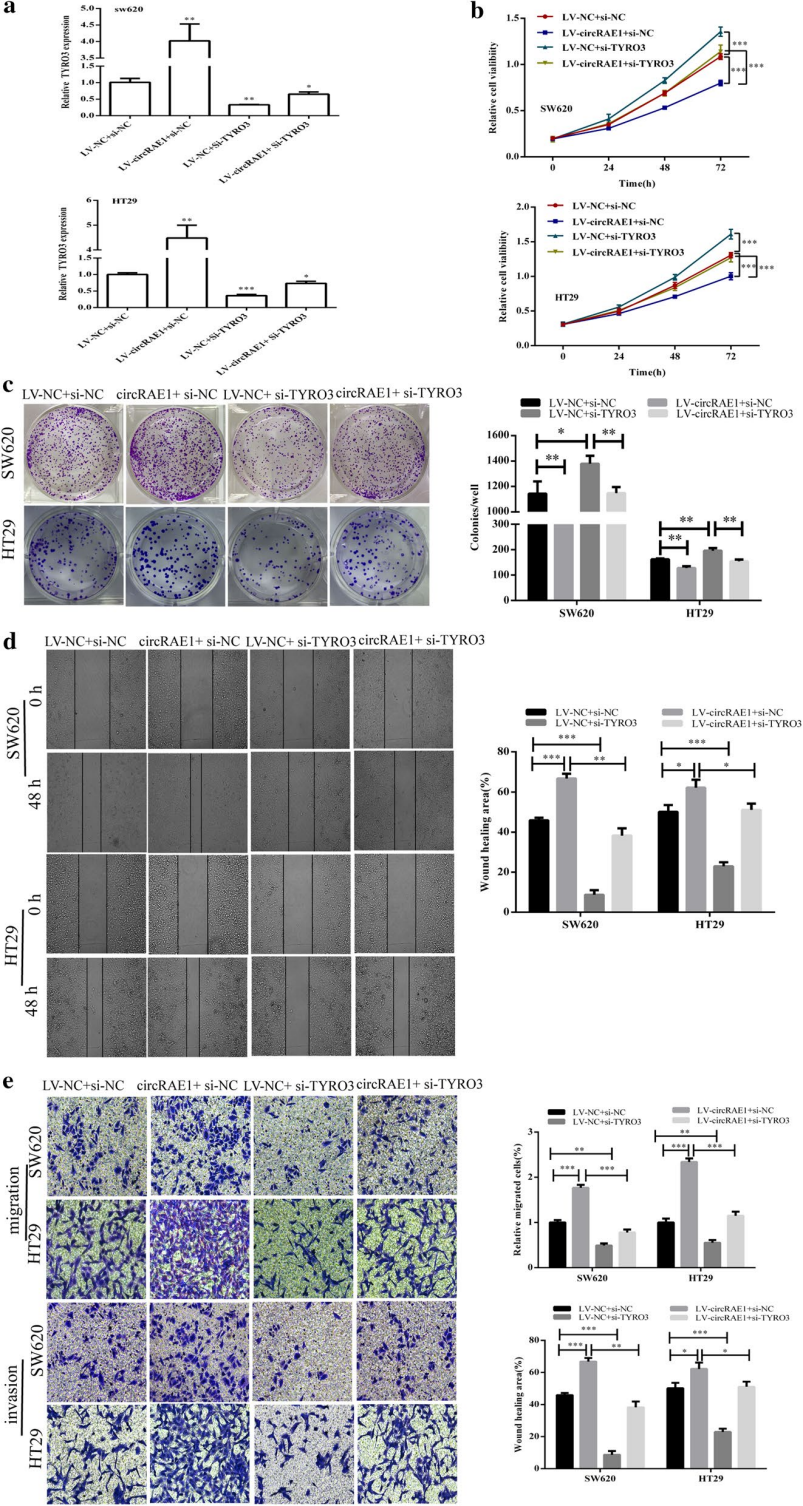
CircRAE1 functions by regulating miR-338-3p/TYRO3 axis in CRC. **a** Rescued effects by Si-TYRO3 of CircRAE1 on TYRO3 expression levels. **b**, **c** Reversed effects by Si-TYRO3 of CircRAE1 upregulation on cell proliferation of SW620 and HT29 cells as determined by CCK8 and colony formation assays. **d** Reversed effects by Si-TYRO3 of CircRAE1 upregulation on the migration activities of SW620 and HT29 cells as determined by wound healing tests. **e** Reversed the effects by Si-TYRO3 of CircRAE1 upregulation on the migration and invasion activities of SW620 and HT29 cells as determined by Transwell assays. *P < 0.05, **P < 0.01, ***P < 0.001

We found that the expression of TYRO3 was upregulated by LV-CircRAE1, and it was significantly reduced by si-TYRO3 (Fig. 6a). Functionally, si-TYRO3 suppressed the proliferation, and the wound closure in the SW620 and HT29 cells and the circRAE1 overexpression partially blocked the effects of si-TYRO3 on both cells (Fig. 6b–d). We also found from the Transwell migration and invasion experiments that si-TYRO3 remarkably reduced the migration and invasion capacity of the SW620 and HT29 cells, and the circRAE1 overexpression could rescue this effect (Fig. 6e). The results suggest that circRAE1 can alter TYRO3 expression to regulate CRC cell migration and invasion.

## Discussion

We validated in this study that circRAE1 was upregulated in CRC tissues, and it was positively associated with the much higher TNM stage, larger tumor size, and more lymph node metastasis in CRC patients. The loss-of-function assays showed that the silencing of circRAE1 could notably reduce the proliferation ability of CRC cells and cell wound closure efficiency, migration and invasion in vitro. The gain-of-function assays showed that the circRAE1 overexpression promoted CRC cell migration and invasion by acting as a ceRNA for miR-388-3p to regulate the TYRO3 levels. These data indicate that circRAE1 can serve as an oncogene in CRC tumorigenesis.

In accordance with remarkable advances in microarray and RNA sequencing technology, the growing numbers of circRNAs have been revealed to play vital roles in numerous disease processes, especially in cancers, including CRC [18]. For instance, circ_0026344 could suppress CRC cell growth and invasion in vitro and reduce CRC growth in vivo by sponging miR-21 and miR-31 [19]. Hsa_circ_0000069, when dramatically upregulated in CRC tissues, could promote proliferation, migration, and invasion of CRC cells [20]. The circRNA hsa_circ_000984 was also found to promote colon cancer growth and metastasis by binding miR-106b to increase CDK6 expression [21]. In this context, by analyzing the circRNA chips of four paired CRC tissues and their adjacent non-tumor control, as previously reported by Chen et al. [17], we further discovered a higher expression of circRAE1 in CRC tissue samples. The qRT-PCR analysis confirmed a similar pattern in CRC cell lines. A high circRAE1 expression indicates bad prognosis of patients with CRC. On the basis of the wound healing tests and Transwell assays, we can conclude that circRAE1 can function as an oncogene to promote CRC cell migration and invasion.

Recently, further research has revealed that circRNAs preferred to function as ceRNAs to sponge various miRNAs, leading to the increase in miRNA target expressions [22]. Hence, we conducted bioinformatics analyses via online (CircInteractome) and uncovered that miR-338-3p could be bound by CircRAE1. Previous studies have shown that miR-338 could suppress tumor progression in various human cancers. Zhang et al. found that miR-338 inhibited bladder cancer cell proliferation and invasion by reducing ETS1 [23]. He et al. confirmed that miR-338 inhibited cell proliferation and the expression of EMT markers of NSCLC cells by directly downregulating the NFATc1 expression [24]. By targeting MACC1, miR-338-3p could inhibit sw480 proliferation and migration and induce apoptosis [25]. Sun et al. demonstrated that miR-338-3p was remarkably downregulated in CRC compared with the adjacent non-tumor tissues [26], a finding that accords with our current results. In the present study, our data indicate that miR-338-3p has an inverse impact on wound healing efficiency and migration and invasion in contrast to circRAE1 in CRC cells. The overexpression of circRAE1 could reverse the effect on CRC cells induced by miR-338-3p mimics and vice versa. We used TYRO3 as a direct target of miR-338-3pin CRC cells to further determine the mechanism of the circRNA–miRNA–mRNA network. TYRO3 is a protein tyrosine kinase. Previous literature has shown that TYRO3 is significantly increased in various cancers, and it promotes cancer cell proliferation and metastasis and enhances drug resistance, and thus is a potential therapeutic target [27, 28]. In addition, TYRO3 was also confirmed to play vital roles in regulating the expression of EMT markers, chemical resistance, liver metastasis, cell proliferation, and apoptosis in CRC as an oncogene [29]. Here, we determined that knocking down the TYRO3 expression could rescue the increased cell wound healing efficiency and the migration and invasion induced by the overexpression of circRAE1 and vice versa; these expressions were also mediated by miR-338 mimics. Nonetheless, further in vivo assays are needed to further confirm the effectiveness in the clinical setting.

## Conclusion

We identified the network consisting of circRAE1, miR-338-3p, and TYRO3 in CRC cells and determined that the increased circRAE1 can serve as an oncogene in CRC cells by functioning as a ceRNA to sponge miR-338-3p, resulting in an upregulated TYRO3 expression. These results can provide a novel potential therapeutic strategy to target the circRAE1/miR-388-3p/TYRO3 axis.

## Data Availability

Not applicable.

## Abbreviations

LncRNAs: long non-coding RNAs
CircRNAs: circular RNAs
CRC: colorectal cancer
EMT: epithelial–mesenchymal transition
qRT-PCR: quantitative RT-PCR assays
WT: the wild-type
MUT: mutant
PFA: paraformaldehyde
ceRNA: competing endogenous RNA

## References

[1] Siegel RL, Miller KD, Jemal A (2018). Cancer statistics, 2018. CA Cancer J Clin.

[2] Taborda MI, Ramírez S, Bernal G (2017). Circular RNAs in colorectal cancer: possible roles in regulation of cancer cells. World J Gastroint Oncol.

[3] Miller KD, Nogueira L, Mariotto AB, Rowland JH (2019). Cancer treatment and survivorship statistics, 2019. CA Cancer J Clin.

[4] Meng S, Zhou H, Feng Z, Xu Z, Tang Y, Li P, Wu M (2017). CircRNA: functions and properties of a novel potential biomarker for cancer. Mol Cancer.

[5] Zhou R, Wu Y, Wang W, Su W, Wang Y, Fan C (2018). Circular RNAs (circRNAs) in cancer. Cancer Lett.

[6] Li Y, Zheng Q, Bao C (2015). Circular RNA is enriched and stable in exosomes: a promising biomarker for cancer diagnosis. Cell Res.

[7] Chen LL, Yang L (2015). Regulation of circRNA biogenesis. RNA Biol.

[8] Su X, Su J, He H, Zhan Y, Liu HC (2019). Hsa_circ_0070269 inhibits hepatocellular carcinoma progression through modulating miR-182/NPTX1 axis. Biomed Pharmacother.

[9] Li F, Zhang L, Li W, Deng J, Zheng J, An MX (2015). Circular RNA ITCH has inhibitory effect on ESCC by suppressing the Wnt/β-catenin pathway. Oncotarget.

[10] Kong R (2020). Circular RNA hsa_circ_0085131 is involved in cisplatin-resistance of non-small cell lung cancer cells by regulating autophagy. Cell Biol Int.

[11] Yu L, Gong X, Sun L, Zhou Q, Lu B, Zhu L (2016). The circular RNA Cdr1as act as an oncogene in hepatocellular carcinoma through targeting miR-7 expression. PLoS ONE.

[12] Chen C, Huang Z, Mo X (2020). The circular RNA 001971/miR-29c-3p axis modulates colorectal cancer growth, metastasis, and angiogenesis through VEGFA. J Exp Clin Cancer Res.

[13] Ma Z, Han C, Xia W (2020). circ5615 functions as a ceRNA to promote colorectal cancer progression by upregulating TNKS. Cell Death Dis.

[14] Yu J, Xu Q, Wang Z, Yang Y, Zhang L, Ma JZ (2018). Circular RNA cSMARCA5 inhibits growth and metastasis in hepatocellular carcinoma. J Hepatol.

[15] Lu YC, Chang JT, Liao CT, Kang CJ, Huang SF, Chen IH (2014). OncomiR-196 promotes an invasive phenotype in oral cancer through the NME4-JNK-TIMP1-MMP signaling pathway. Mol Cancer.

[16] Chiu CC, Lin CY, Lee LY, Chen YJ, Lu YC, Wang HM (2011). Molecular chaperones as a common set of proteins that regulate the invasion phenotype of head and neck cancer. Clin Cancer Res.

[17] Chen S, Zhang L, Su Y, Zhang X (2018). Screening potential biomarkers for colorectal cancer based on circular RNA chips. Oncol Rep.

[18] Li XN, Wang ZJ, Ye CX, Zhao BC, Li ZL, Yang Y (2018). RNA sequencing reveals the expression profiles of circRNA and indicates that circDDX17 acts as a tumor suppressor in colorectal cancer. J Exp Clin Cancer Res.

[19] Yuan Y, Liu W, Zhang Y, Zhang Y, Sun S (2018). CircRNA circ_0026344 as a prognostic biomarker suppresses colorectal cancer progression via microRNA-21 and microRNA-31. Biochem Biophys Res Commun.

[20] Guo JN, Li J, Zhu CL, Feng WT, Shao JX, Wan L (2016). Comprehensive profile of differentially expressed circular RNAs reveals that hsa_circ_0000069 is upregulated and promotes cell proliferation, migration, and invasion in colorectal cancer. OncoTargets Ther.

[21] Xu XW, Zheng BA, Hu ZM, Qian ZY, Huang CJ, Liu XQ, Wu WD (2017). Circular RNA hsa_circ_000984 promotes colon cancer growth and metastasis by sponging miR-106b. Oncotarget.

[22] Bonizzato A, Gaffo E, Te Kronnie G, Bortoluzzi S (2016). CircRNAs in hematopoiesis and hematological malignancies. Blood Cancer J.

[23] Zhang R, Shi H, Ren F, Feng W, Cao Y, Li G (2019). MicroRNA-338-3p suppresses ovarian cancer cells growth and metastasis: implication of Wnt/catenin beta and MEK/ERK signaling pathways. J Exp Clin Cancer Res.

[24] He W, Lu J (2019). MiR-338 regulates NFATc1 expression and inhibits the proliferation and epithelial-mesenchymal transition of human non-small-cell lung cancer cells. Mol Genet Genomic Med.

[25] Lu M, Huang H, Yang J, Li J, Zhao G, Li W (2019). miR-338-3p regulates the proliferation, apoptosis and migration of SW480 cells by targeting MACC1. Exp Ther Med.

[26] Sun K, Su G, Deng H, Dong J, Lei S, Li G (2014). Relationship between miRNA-338-3p expression and progression and prognosis of human colorectal carcinoma. Chin Med J (Enql).

[27] Smart SK, Vasileiadi E, Wang X, Deryckere D, Graham DK (2018). The Emerging Role of TYRO3 as a therapeutic target in cancer. Cancers.

[28] Qin A, Qian W (2018). MicroRNA-7 inhibits colorectal cancer cell proliferation, migration and invasion via TYRO3 and phosphoinositide 3-kinase/protein B kinase/mammalian target of rapamycin pathway suppression. Int Mol Med.

[29] Chien CW, Hou PC, Wu HC, Chang YL, Lin SC, Lin BW (2016). Targeting TYRO3 inhibits epithelial-mesenchymal transition and increases drug sensitivity in colon cancer. Oncogene.

